# Prevalence and related factors of physical function and cognitive impairment among older adults: a population-based regional cross-sectional study

**DOI:** 10.3389/fnagi.2025.1534824

**Published:** 2025-03-14

**Authors:** Yi Zhang, Guifen Cheng, Ling Chen, Xiaoxia Wang, Lixia Lin, Qiao Huang, Jinhua Guo, Bei Gong, Tiemei Shen

**Affiliations:** ^1^Guangdong Cardiovascular Institute, Guangdong Provincial People's Hospital, Guangdong Academy of Medical Sciences, Southern Medical University, Guangzhou, Guangdong, China; ^2^Survice Supervision Center, Guangdong Provincial People's Hospital, Guangdong Academy of Medical Sciences, Southern Medical University, Guangzhou, Guangdong, China; ^3^Guangdong Geriatric Institute, Guangdong Provincial People's Hospital, Guangdong Academy of Medical Sciences, Southern Medical University, Guangzhou, Guangdong, China; ^4^Department of Nursing, Guangdong Provincial People's Hospital, Guangdong Academy of Medical Sciences, Southern Medical University, Guangzhou, Guangdong, China

**Keywords:** physical function, cognitive impairment, older adults, a population-based regional cross-sectional study, prevalence and related factors

## Abstract

**Background and aims:**

As the country with the largest and fastest-aging older population worldwide, China has hosted an increasing number of regional investigations into disability among older adults. However, the prevalence of disabilities related to physical function and cognition in southern China remains unknown. This study aimed to assess the prevalence of and associated factors for cognitive and physical function impairment in individuals aged 60 years and older.

**Methods:**

For this population-based cross-sectional study, a total of 5,603 participants were recruited between June 2021 and December 2022 using a multistage, stratified, cluster sampling procedure. Instruments, including a general questionnaire, basic and instrumental activities of daily living, the Chinese version of the Mini-Mental State Examination (MMSE), the Patient Health Questionnaire-9 (PHQ-9), and the Generalized Anxiety Disorder-7 (GAD-7), were used to collect data through a WeChat mini program. Binary and multivariate logistic regression analyses were applied to explore the influencing factors.

**Results:**

The prevalence of physical function and cognitive impairment among older adults was 37.3 and 31.0%, respectively. Multivariate regression analyses revealed that age, family income, education level, place of residence, medication type, annual physical examinations, weekly social activities, support from family or friends, hearing disorders, walking disorders, and depression were all associated with both physical function and cognitive impairment. Moreover, an increased risk of physical function impairment correlated with BMI, region, income source, smoking, and weekly exercise, while cognitive impairment was associated with the number of children, insurance type, coronary heart disease, and anxiety. Physical function (*OR*: 1.79, 95% *CI*: 1.49–2.16) and cognitive impairment (*OR*: 1.83, 95% *CI*: 1.51–2.21) were mutually influential in our study.

**Conclusion:**

This study showed a high prevalence of various factors related to physical function and cognitive impairment. The results revealed that comprehensive and systematic prevention and control programs for disabilities should be developed to improve the quality of life for older adults.

## 1 Introduction

With the growing trend of an aging population, China has gradually come to be regarded as having the largest elderly population worldwide, resulting in a substantial treatment-related economic burden for families and the healthcare system (Qiao et al., 2022; Han et al., 2022). According to the 7th national census in 2020, there were 26,402 million people aged 60 years or older in China, accounting for 18.7% of the total population, which was 5.4% higher than in 2010 (Statistic N B O, 2021). As a populous country experiencing rapid and significant aging, the health status of elderly individuals is concerning (Damluji et al., 2021; Han et al., 2022). Older adults face various physical and mental health issues, leading to lengthy durations of illness and a significant increase in the number of older adults with disabilities. Wang and Li (2020) reported that the estimated proportion of disabled older adults within the total population is rising year by year and is expected to reach 13.7% by 2050. Disability status not only affects the overall quality of life among older adults but also contributes to a greater demand for long-term care and associated costs, ultimately posing a substantial challenge to social care and medical security (Hsieh and Waite, 2019; Liao et al., 2022; Zhang et al., 2022). Therefore, exploring the relationship between disability status and chronic diseases, the mechanisms and risk factors contributing to disability, and the establishment of prevention and treatment systems for disabilities has gradually become an important research topic in gerontology.

Disability status is a fundamental measure for assessing the functional capacity and health level of older adults (Wang and Li, 2020). The World Health Organization (WHO) and the Chinese disability classification standard define disability to encompass physical, visual, hearing, phonological, and cognitive impairment (Abdi et al., 2019).

The functional state used to define physical disability typically consists of activities of daily living (ADL), muscle strength, balance, and gait speed (Rajan et al., 2012). ADL assessment—which includes basic activities of daily living (BADL) and instrumental activities of daily living (IADL)—is regarded as a primary aspect widely used in previous studies (Wang et al., 2023; Rajan et al., 2012) to determine physical function or self-care ability among older adults due to its relatively simple and convenient nature.

BADLs refer to basic self-care activities, such as eating and dressing, that fulfill essential physiological requirements, while IADLs include more complex tasks that arise later and typically have a shorter duration than BADLs (Gold, 2012; Cornelis et al., 2019).

Cognitive impairment leading to dementia is a serious global public health concern, influenced by the growing number of older adults and recognized as a vital indicator for assessing disability (Inocian and Patalagsa, 2016). Mild cognitive impairment (MCI) represents an intermediate stage between normal cognition and dementia, and a growing body of research focused on risk management and interventions has focused on these individuals to prevent dementia (Tangalos and Petersen, 2018; Campbell et al., 2013). In the past 3 years, several studies have been conducted on the current circumstances of older adults with MCI or dementia, with estimated prevalence ranging from 1.2 to 23.2% (Xue et al., 2022; Teh et al., 2021; Liu et al., 2021; Vlachos et al., 2020) and nearly 15% in China (Deng et al., 2021; Jia et al., 2020).

Previous population-based regional studies investigated factors related to physical or cognitive function, such as age (Xue et al., 2022; Chen et al., 2018; Choi et al., 2019), education level (Xue et al., 2022; Jia et al., 2020; Goswami et al., 2019; Ravi et al., 2022), place of residence (Deng et al., 2021; Cheng et al., 2023; Chuang et al., 2021), illness state (Xue et al., 2022; Liu et al., 2021; Jia et al., 2020; Fuller-Thomson et al., 2022; Deckers et al., 2017), and mental problems (Freire et al., 2017; Desai et al., 2021; Kang et al., 2017). However, due to subjective characteristics or measuring approaches, there are still some controversies regarding the factors influencing disability. Moreover, these factors consistently change with social development, lifestyle, and regional environment.

China has the largest population of disabled and semi-disabled older adults in the world due to the increasing rate of deep aging. It is particularly important to explore and establish a long-term care service model and to conduct health function assessments for older adults with disabilities, from family and community settings to professional institutions, in order to promote long-term care services. There is a significant developmental gap between urban and rural areas in China, leading to major discrepancies in functional status among older adults in various institutions, including hospitals, communities, and nursing homes for the elderly. Surveys that capture regional characteristics regarding functional status and related factors among older adults are crucial to meeting diverse service needs. Although an increasing number of regional surveys targeting the prevalence of and factors influencing older adults with disabilities have been conducted nationwide in recent years, little is known about the prevalence of disability in the southern region. Furthermore, the definition of disability in most studies has been based on only a single aspect.

In view of the aforementioned variable factors related to disability and aiming to develop a more comprehensive understanding of the concept, we conducted a large cross-sectional study involving adults aged 60 years or older from approximately six cities in Guangdong Province, China. The aim was to explore the prevalence, associations, and influencing factors of disability related to physical function and cognitive impairment in older adults.

The present study’s hypotheses are as follows: First, there is a high incidence of physical or cognitive impairment in southern China. Second, both physical and cognitive impairments are associated with several factors, including sociodemographic, disease-related, behavior-related, and psychological factors. Finally, there is a significant relationship between physical and cognitive impairments.

## 2 Methods

### 2.1 Study design

We conducted a population-based observational cross-sectional study using a multistage, stratified, cluster-sampling procedure from June 2021 to December 2022.

### 2.2 Participants

The selection of study sites was divided into three stages. First, we selected 1–2 representative cities from the northern, southern, western, and eastern regions of Guangdong Province. Second, we chose 2–3 well-known or representative tertiary hospitals and one secondary hospital in the geriatric field. Given the significant discrepancy in functional states among hospitalized and non-hospitalized older adults, we included only those subjects who regularly attended outpatient follow-ups or utilized daily healthcare services. Third, we selected one community healthcare center and 60–70 families based on the community resident health files. Finally, we included 5,603 adults aged 60 years or older from 15 tertiary hospitals, six secondary hospitals, six community healthcare centers, and 350 families across six cities (Guangzhou, Shenzhen, Zhuhai, Foshan, Maoming, and Qingyuan). The inclusion criteria for participants were as follows: (1) aged≥60 years, (2) able to communicate normally without barriers, and (3) having provided informed consent for voluntary participation in this study. The exclusion criteria included: (1) having mental disorders or a history of mental disorders (e.g., depression and schizophrenia), (2) a diagnosis of dementia, (3) any acute diseases (e.g., myocardial infarction, and stroke), and (4) any conditions leading to limb movement disorders (e.g., trauma, surgery, and musculoskeletal diseases).

### 2.3 Measurements

#### 2.3.1 General questionnaire

This questionnaire was developed by researchers based on related previous studies (Qiao et al., 2022; Chen et al., 2018; Ding et al., 2015; Qin et al., 2022; Ramadass et al., 2018), and research content and was reviewed by two experts, Cui and Chen, prior to the pilot study. The evaluation of general information consisted of four main domains: (1) Demographic characteristics: age, sex, BMI, number of children, and more; (2) Behavioral habits: smoking, drinking, annual physical examinations, weekly social activities, and weekly exercise; (3) Family or social support: assistance from family or care from friends; and (4) Disease-related characteristics: cardiovascular or cerebrovascular diseases, hypertension, coronary heart disease, diabetes, type of medication, hearing disorders, vision disorders, and walking impairments. Participants were asked to indicate whether hearing, vision, or walking had an impact on the daily life of older adults, with three options: no effect, less effect, and obvious effect. The responses of “less effect” or “obvious effect” were deemed indicators of functional impairment in hearing, vision, or walking.

#### 2.3.2 Physical function impairment measurement

This was evaluated using an assessment of activities of daily living (ADLs). ADLs are defined as the essential activities required for daily life, reflecting the fundamental functions of individuals in medical institutions, communities, and families (Cornelis et al., 2019; De Vriendt et al., 2021). The assessment of ADLs is divided into basic activities of daily living (BADLs) and instrumental activities of daily living (IADLs). BADLs pertain to the critical movements and self-care tasks performed in hospitals or at home, which include eight items: eating, bathing, combing, dressing, controlling urination, managing excretion, walking, and ascending and descending stairs (Cornelis et al., 2019). IADLs refer to more complex activities than BADLs, such as those necessitating advanced skills and the use of tools performed within communities. These activities include seven items: shopping, cycling or riding, cooking, doing housework, washing clothes, making phone calls, and taking medication (De Vriendt et al., 2021; Bruderer-Hofstetter et al., 2022).

Each category is assessed on three levels: no assistance, partial assistance, and full assistance. Responses indicating anything other than “no assistance” for each category suggest functional impairment, which is considered a disability.

#### 2.3.3 Cognitive impairment measurement

Cognitive impairment was evaluated using the Chinese version of the Mini-Mental State Examination (MMSE), which has received authorization for its use (Wang and Zhang, 1989). This scale consists of 30 items across four dimensions: orientation, memory, attention and calculation, recall, and language. The sensitivity of this assessment for screening cognitive impairment reached up to 92.5% (Bo et al., 2018). This is the most common and widely used assessment worldwide, designed by Folstein in 1975 (Cockrell and Folstein, 1988). Each correct answer earns one point, while an incorrect or unclear response earns no points. The maximum score on the scale is 30 points, with lower scores indicating more severe cognitive impairment. The cutoff points for cognitive impairment were calculated based on education level: ≤19 points for illiterate individuals, ≤22 points for those with a primary school education, and ≤ 26 points for individuals with a secondary school education or above.

#### 2.3.4 Depressive symptoms

These symptoms were assessed using the Patient Health Questionnaire-9 (PHQ-9). This questionnaire was used to assess the frequency of nine conditions over the past 2 weeks: displeasure, appetite changes, fatigue, feelings of worthlessness, guilt, decreased concentration, slow movements, restlessness, and suicidal tendencies (Kroenke et al., 2001). Each item is rated on a 4-point Likert scale, with total scores ranging from 0 to 27. Depressive symptoms are categorized into four levels: mild (5–9), moderate (10–14), moderate–severe (15–19), and severe (20–27).

#### 2.3.5 Anxiety

This was measured using the Generalized Anxiety Disorder-7 (GAD-7) questionnaire. This tool assessed the frequency of seven conditions over the 2 weeks: tension, uncontrollable worries, excessive worries, an inability to relax, akathisia, irritability, and foreboding (Löwe et al., 2008). Each question is evaluated on a 4-point Likert scale, with total scores ranging from 0 to 21. Depressive symptoms are categorized into four levels: mild (5–9), moderate (10–13), moderate–severe (14–18), and severe (19–21).

### 2.4 Ethics declarations

All procedures conducted in studies involving human participants were in accordance with the ethical standards of the institutional and/or national research committee, the 1964 Helsinki Declaration and its later amendments, or comparable ethical standards. The ethics committee of Guangdong Province People’s Hospital (KY-Z-2021-690-01). Informed consent was obtained from all individual participants.

### 2.5 Data collection procedures

Data collection was conducted using the WeChat mini program called “Jingyice platform for the functional assessment of older adults.” First, we contacted the relevant leaders of selected hospitals and communities to obtain permission for the investigation. Specialized interviewers assigned to each hospital and community were responsible for collecting information in their specific research areas.

A door-to-door survey of families was conducted by interviewers from the respective communities. To ensure a uniform investigation process, online training on questionnaire interpretation and methods was organized, along with a preliminary survey held prior to the formal survey. Interviewers followed standardized instructions to present the study’s objectives and content and engaged in one-on-one, face-to-face dialogue to gather information after obtaining informed consent. Inquiries about family members or caregivers were allowed if participants were unable to communicate directly with interviewers due to speech or hearing disorders. Each data point was securely stored in a file accessible only to authorized personnel.

### 2.6 Statistical analysis

SPSS software version 26.0 was used to analyze the data. Descriptive statistics were employed to identify the distribution of the included factors. Means and standard deviations were used to describe continuous variables, while absolute values and percentages were used to express categorical variables. Independent-sample *t*-tests and chi-square tests were conducted to compare sex differences across each continuous and categorical variable, respectively. Univariate analysis was conducted through bivariate logistic regression to determine whether independent variables, including demographic characteristics, behavioral habits, social support, and disease-related factors, were associated with physical function impairment and cognitive impairment, with estimated odds ratios and 95% confidence intervals provided.

Multivariate logistic regression analysis was conducted to identify the influencing factors, treating the aforementioned variables as independent variables and physical-function impairment or cognitive impairment as dependent variables. A two-sided *p-value* < 0.05 was considered to indicate statistical significance.

## 3 Results

### 3.1 General characteristics

A total of 5,603 adults aged 60 years and older were enrolled in this survey, of whom 2,675 (47.4%) were men and 2,946 (52.6%) were women. The average age of the participants was 71.38 ± 7.65 old. The majority of the older adults lived in Guangdong Province during the survey, including 3,356 (59.9%) subjects living in Guangzhou or Shenzhen and 2,100 (37.5%) participants living in other cities within Guangdong.

The largest proportion of participants in this survey came from the rural population, which accounted for 41.5% of the total. Different levels of anxiety and depression symptoms were observed in 28.2 and 20.5% of subjects, respectively. A total of 2,089 participants aged 60 years or older exhibited a decline in physical function as measured via BADLs and IADLs, with a prevalence of 37.3%. Cognitive impairment, evaluated using the MMSE, was found in 1,378 participants aged 60 years or older, with a prevalence of 31.0%.

There was a significant difference between the sexes, including factors such as age, number of children, religion, living arrangements with children, monthly family income, source of income, education level, marital status, type of insurance, hypertension, coronary heart disease, alcohol consumption, smoking, care from family or friends, vision, walking ability, physical function impairment, and cognitive impairment. The general characteristics of the participants and the prevalence of disability are shown in Table 1.

**Table 1:** General characteristics of the included participants (*n* = 5,603).

Variables and subgroups	Mean ± SD	n	%
Sex			
Male		2,657	47.4
Female		2,946	52.6
Age, years old [Mean (Std)]	71.38(7.65)		
BMI, kg/m^2^ [Mean (Std)]	23.01(3.59)		
Quantity of children [Mean (Std)]	2.97(1.43)		
Region			
Other cities in Guangdong		3,356	59.9
Guangzhou or Shenzhen		2,100	37.5
Other provinces or cities		147	2.6
Religion			
Yes		448	8.0
No		5,155	92.0
Living with children			
Yes		4,091	73.0
No		1,512	27.0
Family income per month,¥			
<2000		1,108	19.8
2000–4,000		2005	35.8
4,000–6,000		1,166	20.8
>6,000		1,324	23.6
Source of income			
Acquiring from children		3,895	69.5
Retirement income		1,312	23.4
Labor income		396	7.1
Education level			
Illiteracy		1,115	19.9
Primary school and below		2,356	42.0
Junior high school		1,125	20.1
Senior high school		811	14.5
College and above		196	3.5
Marital status			
With partner		4,577	81.7
No partner		1,026	18.3
Place of residence			
Villages		2,326	41.5
Counties or towns		855	15.3
Small or middle-sized cities		777	13.9
Large-sized cities		1,645	29.4
Type of insurance			
Rural medical service		3,452	61.6
Urban medical service		1780	31.8
Free medical service		371	6.6
Cardiovascular or cerebrovascular diseases			
Yes		3,270	58.4
No		2,333	41.6
Hypertension			
Yes		2,323	41.5
No		3,280	58.5
Coronary heart disease			
Yes		962	17.2
No		4,641	82.8
Diabetes			
Yes		1,060	18.9
No		4,543	81.1
Type of medication			
None		2,155	38.5
1		1,077	19.2
2		1,093	19.5
3		585	10.4
>4		693	12.4
Drinking			
Never		4,450	79.4
Occasional		547	9.8
Always		606	10.8
Smoking			
Never		3,980	71.0
Occasional		760	13.6
Always		863	15.4
Annual physical examination for the last 10 years			
Yes		3,126	55.8
No		2,477	44.2
Weekly social activities, days			
Never		2,313	41.3
1–3		1,531	27.3
4–6		228	4.1
Every day		1,531	27.3
Weekly exercise, days			
Never		1942	34.7
1–3		1,350	24.1
4–6		243	4.3
Every day		2068	36.9
Support from family			
Yes		3,042	54.3
No		2,561	45.7
Care from family or friends			
Yes		4,570	81.6
No		1,033	18.4
Anxiety			
None		4,058	72.4
Mild		1,245	22.2
Moderate		162	2.9
Moderate–severe		100	1.8
Severe		38	0.7
Depression			
None		4,025	75.0
Mild		1,010	18.0
Moderate		218	3.9
Moderate–severe		115	2.1
Severe		55	1.0
Hearing			
Normal		1705	30.4
Abnormal		3,898	69.6
Vision			
Normal		3,618	64.6
Abnormal		1985	35.4
Walking			
Normal		3,790	67.6
Abnormal		1813	32.4
Physical function impairment			
Yes		2089	37.3
No		3,514	62.7
Cognitive impairment			
Yes		1,378	24.6
No		4,225	75.4

Data are shown by *n* and % except for age, BMI, and number of children.

BMI = body mass index.

### 3.2 Influencing factors associated with physical function impairment

**Figure 1:**
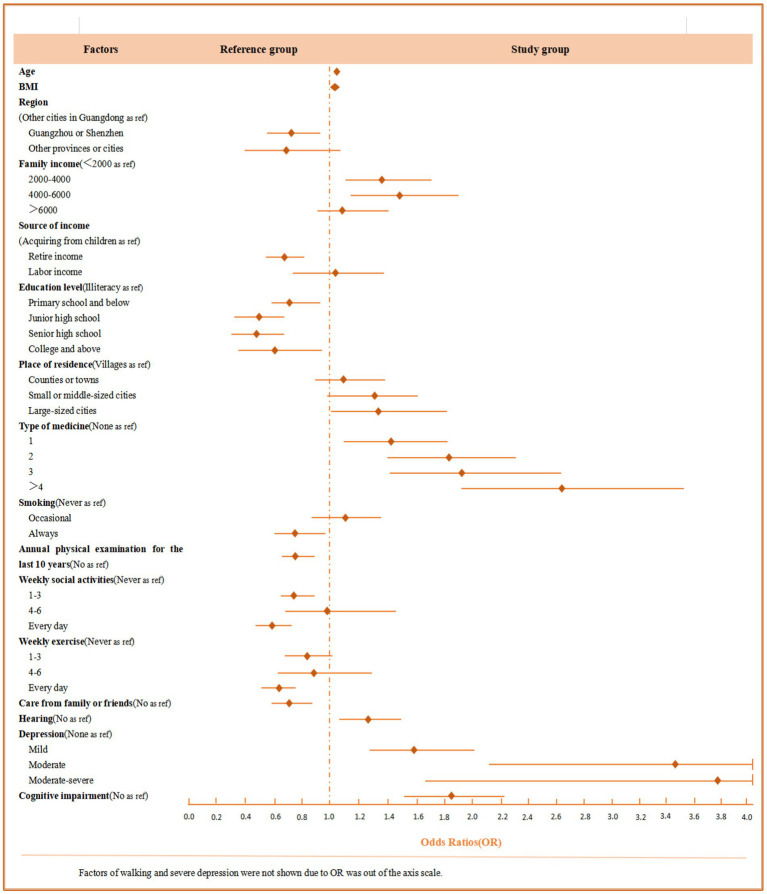
Odds ratios (*OR*) and 95% confidence intervals (95% *CI*) for factors that are statistically significant in relation to physical function impairment.

As shown in Table 2, variables extracted through univariate analysis with statistical significance were included to establish a multivariate logistic regression. The results indicated that older age (*OR*: 1.06, 95% *CI*: 1.05, 1.07) and a higher BMI (*OR*: 1.03, 95% *CI*: 1.01, 1.05) were associated with an increased risk of physical function impairment. Older adults living in other cities in Guangdong, receiving income from children, being illiterate, and residing in large cities faced a higher risk of physical function impairment. A family monthly income between 2000 and 4,000¥ (*OR*: 1.40, 95% *CI*: 1.13, 1.72) or between 4,000 and 6,000¥ (*OR*: 1.51, 95% *CI*: 1.18, 1.92) was linked to an increased risk of physical function impairment. Regarding behavioral habits, older adults who had never smoked, had not received an annual physical examination in the past 10 years, and did not participate in weekly social activities were more likely to experience physical function impairment. In terms of family or social support, older adults not cared for by family or friends were at a greater risk of physical function impairment. For disease-related characteristics, taking multiple types of medication, having hearing disorders (*OR*: 1.24, 95% *CI*: 1.09, 1.43), and having impaired walking ability (*OR*: 6.35, 95% *CI*: 5.39, 7.45) were also correlated with a higher risk of physical function impairment. For psychological factors, older adults suffering from mild (*OR*: 1.58, 95% *CI*: 1.52, 2.00), moderate (*OR*: 3.46, 95% *CI*: 2.13, 5.61), or moderate–severe (*OR*: 3.94, 95% *CI*: 1.70, 9.12) depression showed an increasing risk of physical function impairment. The multivariate logistic regression analysis of risk factors for physical impairment among adults aged 60 years or older is presented in Table 3 and Figure 1.

**Table 2:** Univariate analysis of influencing factors for physical function or cognitive impairment (*n* = 5,603).

Variables and subgroups	No disability (*n* = 3,514)	Disability (*n* = 2089)	*OR* (95%*CI*)	No cognitive Impairment (*n* = 4,225)	Cognitive Impairment (*n* = 1,378)	*OR* (95%*CI*)
Sex						
Male	1728 (49.2)	929 (44.5)	1.21 (1.08, 1.35)**	1955 (46.3)	702 (50.9)	0.83 (0.73, 0.94)**
Female	1786 (50.8)	1,160 (55.5)		2,270 (53.7)	676 (49.1)	
Age, years old [Mean (Std)]	69.20 (6.10)	75.05 (8.53)	1.12 (1.11, 1.13)**	70.97 (7.25)	72.64 (8.64)	1.03 (1.02, 1.04)**
BMI, kg/m^2^ [Mean (Std)]	22.92 (3.50)	23.25 (3.75)	1.02 (1.00, 1.03)*	22.99 (3.63)	23.03 (3.47)	1.00 (0.99, 1.02)
Quantity of children [Mean (Std)]	2.80 (1.31)	3.24 (1.57)	1.24 (1.19, 1.29)**	2.99 (1.42)	2.91 (1.48)	0.96 (0.92, 1.01)
Region						
Other cities in Guangdong	1935 (55.1)	1,421 (68.0)	0.56 (0.50, 0.63)**	2,552 (60.4)	804 (58.4)	1.03 (0.93, 1.16)
Guangzhou or Shenzhen	1,486 (42.3)	614 (29.4)		1,551 (36.7)	549 (39.8)	
Other provinces or cities	93 (2.6)	54 (2.6)		122 (2.9)	25 (1.8)	
Religion						
Yes	273 (7.8)	175 (8.4)	1.09 (0.89, 1.32)	344 (8.1)	104 (7.6)	0.92 (0.73, 1.16)
No	3,241 (92.2)	1914 (91.6)		3,881 (91.9)	1,274 (92.4)	
Living with children						
Yes	2,609 (74.3)	1,482 (70.9)	0.85 (0.75, 0.96)**	3,129 (74.1)	962 (69.8)	0.81 (0.71, 0.93)**
No	905 (25.7)	607 (29.1)		1,096 (25.9)	416 (30.2)	
Family income per month,¥						
<2000	672 (19.1)	436 (20.9)	0.88 (0.84, 0.93)**	815 (19.3)	293 (21.3)	0.91 (0.86, 0.96)**
2000–4,000	1,203 (34.2)	802 (38.4)		1,481 (35.1)	524 (38.0)	
4,000–6,000	721 (20.5)	445 (21.3)		893 (21.1)	273 (19.8)	
>6,000	918 (26.2)	406 (19.4)		1,036 (24.5)	288 (20.9)	
Source of income						
Acquiring from children	2,327 (66.2)	1,568 (75.1)	0.55 (0.48, 0.63)**	2,990 (70.8)	905 (65.7)	1.18 (1.07, 1.30)**
Retirement income	957 (27.2)	355 (16.9)		950 (22.5)	362 (26.3)	
Labor income	230 (6.6)	166 (8.00)		285 (6.7)	111 (8.0)	
Education level						
Illiteracy	534 (15.2)	581 (27.8)	0.59 (0.51, 0.68)**	955 (22.6)	160 (11.6)	1.59 (1.50, 1.68)**
Primary school and below	1,440 (40.9)	916 (43.9)		1981 (46.9)	375 (27.2)	
Junior high school	807 (22.9)	318 (15.2)		637 (15.1)	488 (35.4)	
Senior high school	604 (17.2)	207(9.9)		524 (12.4)	287 (20.9)	
College and above	129(3.8)	67 (3.2)		128 (3.0)	68 (4.9)	
Marital status						
With partner	3,043 (86.6)	1,534 (73.4)	2.34 (2.04, 2.68)**	3,443 (81.5)	1,134 (82.3)	0.95 (0.81, 1.11)
No partner	471 (13.4)	555 (26.6)		782 (18.5)	244 (17.7)	
Place of residence						
Villages	1,390 (39.6)	936 (44.8)	0.86 (0.82, 0.89)**	1827 (43.2)	499 (36.2)	1.13 (1.07, 1.18)**
Counties or towns	489 (13.9)	366 (17.5)		658 (15.6)	197 (14.3)	
Small or middle-sized cities	459 (13.1)	318 (15.2)		542 (12.8)	235 (17.1)	
Large cities	1,176 (33.5)	469 (22.5)		1,198 (28.4)	447 (32.4)	
Type of insurance						
Rural medical service	2,164 (61.6)	1,288 (61.7)	0.99 (0.91, 1.09)	2,691 (63.7)	761 (55.2)	1.28 (1.16, 1.41)**
Urban medical service	1,115 (31.7)	665 (31.8)		1,266 (29.9)	514 (37.3)	
Free medical service	235 (6.7)	136 (6.5)		268 (6.4)	103 (7.5)	
Cardiovascular or cerebrovascular diseases						
Yes	1786 (50.8)	1,484 (71.0)	2.37 (2.12, 2.66)**	2,363 (55.9)	907 (65.8)	1.52 (1.34, 1.72)**
No	1728 (49.2)	605 (29.00)		1862 (44.1)	471 (34.2)	
Hypertension						
Yes	1,222 (34.8)	1,101 (52.7)	2.09 (1.87, 2.33)**	1,686 (39.9)	637 (46.2)	1.30 (1.15, 1.46)**
No	2,292 (65.2)	988 (47.3)		2,539 (60.1)	741 (53.8)	
Coronary heart disease						
Yes	447 (12.7)	515 (24.7)	2.25 (1.95, 2.58)**	660 (15.6)	302 (21.9)	1.52 (1.30, 1.77)**
No	3,067 (87.3)	1,574 (75.3)		3,565 (84.4)	1,076 (78.1)	
Diabetes						
Yes	552 (15.7)	508 (24.3)	1.72 (1.51, 1.97)**	757 (17.9)	303 (22.0)	1.29 (1.11, 1.50)**
No	2,962 (84.3)	1,581 (75.7)		3,468 (82.1)	1,075 (78.0)	
Type of medication						
None	1,677 (47.7)	478 (22.9)	1.54 (1.47, 1.60)**	1709 (40.4)	446 (32.4)	1.13 (1.08, 1.18)**
1	688 (19.6)	389 (18.6)		782 (18.5)	295 (21.4)	
2	599 (17.1)	494 (23.7)		836 (19.80)	257 (18.7)	
3	282 (8.0)	303 (14.5)		420 (9.9)	165 (11.9)	
>4	268(7.6)	425(20.3)		478(11.2)	215(15.6)	
Drinking						
Never	2,774 (78.9)	1,676 (80.2)	0.85 (0.78, 0.93)**	3,346 (79.2)	1,104 (80.1)	0.94 (0.86, 1.04)
Occasional	288 (8.2)	259 (12.4)		406 (9.6)	141 (10.2)	
Always	452 (12.9)	154 (7.4)		473 (11.2)	133 (9.7)	
Smoking						
Never	2,475 (70.4)	1,505 (72.0)	0.84 (0.78, 0.91)**	2,992 (70.8)	988 (71.7)	0.95 (0.87, 1.03)
Occasional	398 (11.3)	362 (17.3)		561 (13.3)	199 (14.4)	
Always	641 (18.2)	222 (10.7)		672 (15.9)	191 (13.9)	
Annual physical examination for the last 10 years						
Yes	1734 (49.4)	743 (35.6)	0.57 (0.51, 0.63)**	1832 (43.4)	645 (46.8)	1.15 (1.02, 1.30)*
No	1780 (50.6)	1,346 (64.4)		2,393 (56.6)	733 (53.2)	
Weekly social activities, days						
Never	1,143 (32.5)	1,170 (56.1)	0.64 (0.61, 0.67)**	1,626 (38.5)	687 (49.9)	0.81 (0.77, 0.86)**
1–3	1,006 (28.6)	525 (25.1)		1,179 (27.9)	352 (25.5)	
4–6	146 (4.2)	82 (3.9)		168 (4.0)	60 (4.4)	
Every day	1,219 (34.7)	312 (14.9)		1,252 (29.6)	279 (20.2)	
Weekly exercise, days						
Never	870 (24.8)	1,072 (51.3)	0.61 (0.59, 0.64)**	1,421 (33.6)	521 (37.8)	0.93 (0.88, 0.97)**
1–3	861 (24.5)	489 (23.4)		1,019 (24.1)	331 (24.0)	
4–6	159 (4.5)	84 (4.0)		176 (4.2)	67 (4.9)	
Every day	1,624 (46.2)	444 (21.3)		1,609 (38.1)	459 (33.3)	
Support from family						
Yes	1933 (55.0)	1,109 (53.1)	0.93 (0.83, 1.03)	2,364 (55.9)	678 (49.2)	0.76 (0.68, 0.86)**
No	1,581 (45.00)	980 (46.9)		1861 (44.1)	700 (50.8)	
Care from family or friends						
Yes	3,035 (86.4)	1,535 (73.5)	0.44 (0.38, 0.50)**	3,529 (83.5)	1,041 (75.5)	0.61 (0.53, 0.71)**
No	479 (13.6)	554 (26.5)		696 (16.5)	337 (24.5)	
Anxiety						
None	2,922 (83.1)	1,136 (54.4)	2.88 (2.61, 3.19)**	3,216 (76.2)	842 (61.1)	1.73 (1.60, 1.88)**
Mild	526 (14.9)	719 (34.4)		859 (20.3)	386 (28.0)	
Moderate	43 (1.2)	119 (5.7)		103 (2.4)	59 (4.3)	
Moderate–severe	21 (0.6)	79 (3.8)		39 (0.9)	61 (4.4)	
Severe	2 (0.2)	36 (1.7)		8 (0.2)	30 (2.2)	
Depression						
None	3,066 (87.3)	1,139 (54.5)	3.51 (3.16, 3.90)**	3,371 (79.8)	834 (60.5)	1.86 (1.72, 2.01)**
Mild	387 (11.0)	623 (29.8)		674 (15.9)	336 (24.4)	
Moderate	43 (1.2)	175 (8.4)		120 (2.8)	98 (7.1)	
Moderate–severe	15 (0.4)	100 (4.8)		46 (1.1)	69 (5.0)	
Severe	3 (0.1)	52 (2.5)		14 (0.4)	41 (3.0)	
Hearing						
Normal	2,816 (80.1)	1,082 (51.8)	3.76 (3.33, 4.23)**	3,053 (72.3)	845 (61.3)	1.64 (1.45, 1.87)**
Abnormal	698 (19.9)	1,007 (48.2)		1,172 (27.7)	533 (38.7)	
Vision						
Normal	2,593 (73.8)	1,025 (49.1)	2.92 (2.61, 3.28)**	2,855 (67.6)	763 (55.4)	1.68 (1.48, 1.90)**
Abnormal	921 (26.2)	1,064 (50.9)		1,370 (32.4)	615 (44.6)	
Walking						
Normal	3,109 (88.5)	681 (32.6)	15.87 (13.82, 18.22)**	3,071 (72.7)	719 (52.2)	2.44 (2.15, 2.77)**
Abnormal	405 (11.5)	1,408 (67.4)		1,154 (27.3)	659 (47.8)	
Cognitive impairment						
Yes	654 (18.6)	724 (34.7)	2.32 (2.05, 2.63)**			
No	2,860 (81.4)	1,365 (65.3)				
Physical function impairment						
Yes				1,365 (32.3)	724 (52.5)	2.32 (2.05, 2.63)**
No				2,860 (67.7)	654 (47.5)	

Data are shown by n and % except for age, BMI, and quantities of children.

OR = odds ratio, 95% CI = 95% confidence interval.

BMI = body mass index.

*p < 0.05, **p < 0.01.

The statistically significant variables are highlighted as bold values.

**Table 3:** Multivariate logistic regression analysis of influencing factors for physical function impairment (*n* = 5,603).

Influencing factors	β (SE)	Adjusted *OR* (*95%*CI)	*p* value
Age	**0.06 (0.01)**	**1.06 (1.05, 1.07)**	<0.001**
BMI	**0.03 (0.01)**	**1.03 (1.01, 1.05)**	0.015*
Quantity of children	0.01(0.03)	1.01(0.97, 1.07)	0.646
Sex	0.07(0.09)	1.08(0.90, 1.29)	0.408
Region			
Other cities in Guangdong	as ref		
Guangzhou or Shenzhen	**−0.31 (0.12)**	**0.73 (0.57, 0.93)**	0.011*
Other provinces or cities	−0.39 (0.24)	0.68 (0.40, 1.07)	0.097
Living with children	0.06 (0.09)	1.06(0.90, 1.26)	0.488
Family income per month,¥			
<2000	as ref		
2000–4,000	**0.32 (0.11)**	**1.38 (1.12, 1.70)**	0.003**
4,000–6,000	**0.39 (0.13)**	**1.48 (1.16, 1.90)**	0.002**
>6,000	0.09 (0.13)	1.10 (0.86, 1.40)	0.467
Source of income			
Acquiring from children	as ref		
Retirement income	**−0.41 (0.10)**	**0.66 (0.55, 0.81)**	<0.001**
Labor income	0.03 (0.15)	1.03 (0.77, 1.38)	0.850
Education level			
Illiteracy	as ref		
Primary school and below	**−0.33 (0.10)**	**0.72 (0.59, 0.88)**	0.001**
Junior high school	**−0.72 (0.13)**	**0.49 (0.37, 0.63)**	<0.001**
Senior high school	**−0.76 (0.15)**	**0.47 (0.35, 0.63)**	<0.001**
College and above	**−0.51 (0.24)**	**0.60 (0.38, 0.97)**	0.036*
Marital status	0.04 (0.10)	1.04 (0.85, 1.27)	0.705
Place of residence			
Villages	as ref		
Counties or towns	0.10 (0.12)	1.11 (0.89, 1.39)	0.369
Small or middle-sized cities	0.23 (0.12)	1.26(0.99, 1.60)	0.056
Large-sized cities	**0.30 (0.15)**	**1.36 (1.01, 1.81)**	0.040*
Cardiovascular or cerebrovascular diseases	−0.16 (0.14)	0.85 (0.64, 1.13)	0.275
Hypertension	0.21 (0.11)	1.23 (0.99, 1.53)	0.059
Coronary heart disease	0.09 (0.11)	1.09 (0.88, 1.36)	0.424
Diabetes	0.02 (0.11)	1.02 (0.82, 1.26)	0.872
Type of medication			
None	as ref		
1	**0.34 (0.13)**	**1.41(1.10, 1.81)**	0.008**
2	**0.60 (0.14)**	**1.82 (1.40, 2.37)**	<0.001**
3	**0.66 (0.16)**	**1.93 (1.41, 2.62)**	<0.001**
>4	**0.96 (0.16)**	**2.62(1.92, 3.57)**	<0.001**
Drinking			
Never	as ref		
Occasional	0.21 (0.14)	1.24(0.95, 1.63)	0.117
Always	−0.03 (0.14)	0.97 (0.74, 1.26)	0.809
Smoking			
Never	as ref		
Occasional	0.07 (0.13)	1.07 (0.84, 1.37)	0.585
Always	**−0.27 (0.13)**	**0.76(0.60, 0.98)**	0.033*
Annual physical examination for the last 10 years	**−0.27 (0.08)**	**0.76(0.65, 0.90)**	0.001**
Weekly social activities, days			
Never	as ref		
1–3	**−0.30 (0.10)**	**0.74 (0.62, 0.90)**	0.002**
4–6	−0.01 (0.20)	0.99 (0.67, 1.45)	0.953
Every day	**−0.53 (0.11)**	**0.59 (0.48, 0.73)**	<0.001**
Weekly exercise, days			
Never	as ref		
1–3	−0.19 (0.10)	0.83 (0.67, 1.01)	0.064
4–6	−0.12 (0.19)	0.89 (0.61, 1.29)	0.526
Every day	**−0.47 (0.10)**	**0.62 (0.51, 0.76)**	<0.001**
Care from family or friends	**−0.34 (0.10)**	**0.71 (0.59,0.87)**	0.001**
Hearing	**0.23 (0.08)**	**1.26 (1.07, 1.49)**	0.006**
Vision	0.11 (0.08)	1.11 (0.95, 1.31)	0.193
Walking	**1.84 (0.08)**	**6.29 (5.34, 7.41)**	<0.001**
Anxiety			
None	as ref		
Mild	0.21 (0.11)	1.23 (0.99, 1.54)	0.059
Moderate	0.38 (0.27)	1.46 (0.86, 2.50)	0.166
Moderate–severe	−0.31 (0.42)	0.74 (0.32, 1.68)	0.466
Severe	0.91 (1.27)	2.48 (0.21, 29.81)	0.473
Depression			
None	as ref		
Mild	**0.47 (0.12)**	**1.59 (1.26, 2.01)**	<0.001**
Moderate	**1.24 (0.25)**	**3.46 (2.13, 5.62)**	<0.001**
Moderate–severe	**1.33 (0.43)**	**3.79 (1.64, 8.74)**	0.002**
Severe	1.49 (1.02)	4.46 (0.60, 33.01)	0.144
Cognitive impairment	**0.61 (0.10)**	**1.83 (1.51, 2.21)**	<0.001**

OR = odds ratio, 95% CI = 95% confidence interval.

BMI = body mass index.

*p < 0.05, **p < 0.01.

The statistically significant variables are highlighted as bold values.

### 3.3 Influencing factors associated with cognitive impairment

**Figure 2:**
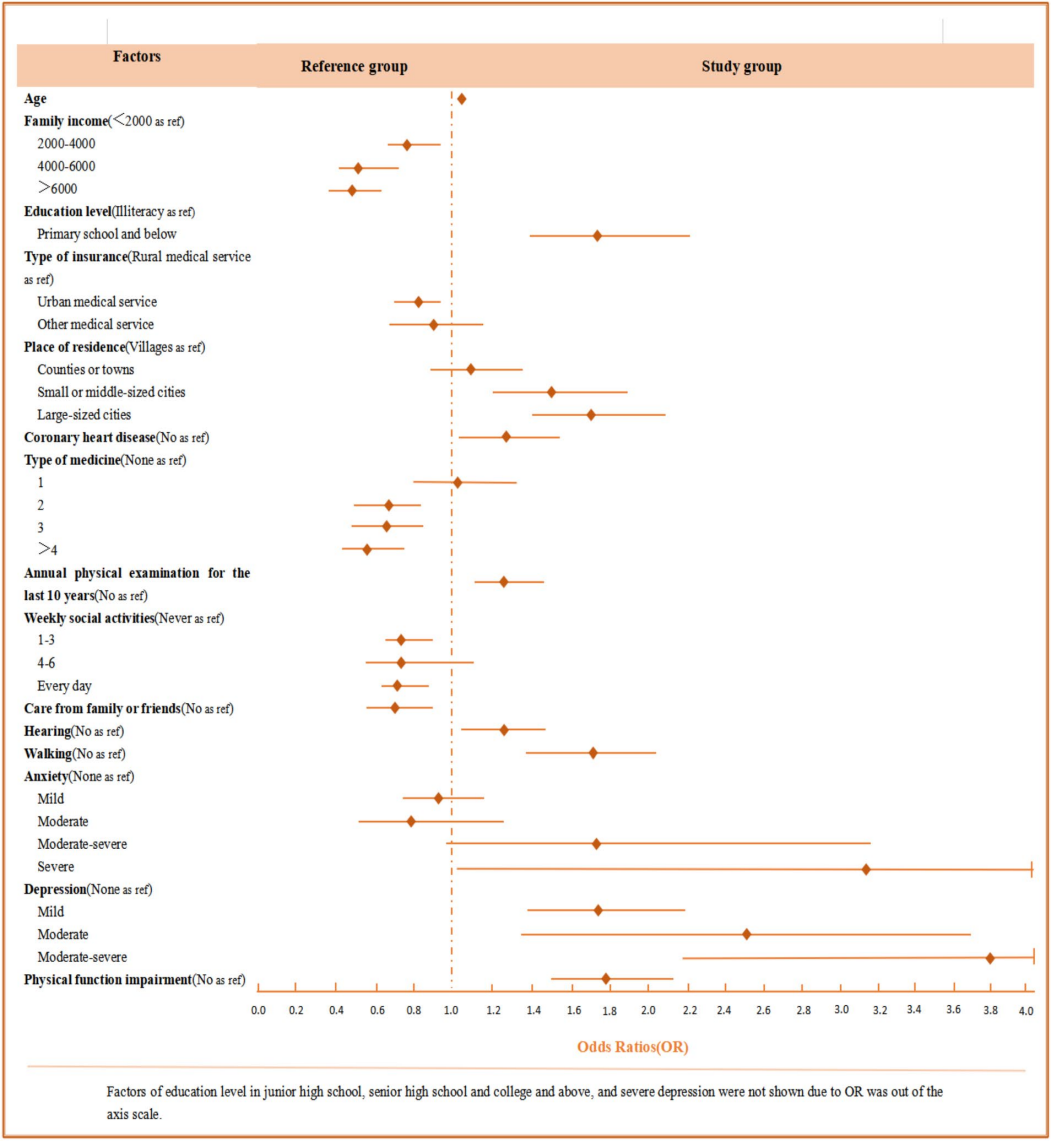
Odds ratios (*OR*) and 95% confidence intervals (95% *CI*) for factors that are statistically significant regarding cognitive impairment.

The results of the multivariate logistic regression analysis of risk factors for cognitive impairment are shown in Table 4 and Figure 2. For demographic characteristics, older age was associated with a higher risk of cognitive impairment (*OR*: 1.02 95% *CI*: 1.01–1.03), as was having fewer children. Education level also played a significant role, with a higher risk observed among those with primary school (*OR*: 1.75, 95%*CI*: 1.40–2.22), junior high school (*OR*: 11.59, 95% *CI*: 8.96–15.00), senior high school (*OR*: 8.71, 95% *CI*: 6.57–11.56), or college education (*OR*: 7.14, 95%*CI*: 4.70–10.86). Additionally, older adults with a family income of <2000¥ and those relying on rural medical services had a higher risk of cognitive impairment. Living in small or middle-sized cities (*OR*: 1.49, 95%*CI*: 1.19–1.87) or large cities (*OR*: 1.68, 95% *CI*: 1.38–2.05) was related to an increased risk compared to those living in villages.

**Table 4:** Multivariate logistic regression analysis of influencing factors for cognitive impairment (*n* = 5,603).

Influencing factors	β (SE)	Adjusted *OR* (*95%*CI)	*p* value
Age	**0.02 (0.01)**	**1.02 (1.01–1.03)**	0.001**
Sex	0.10 (0.07)	1.11 (0.96–1.28)	0.159
Living with children	−0.06 (0.08)	0.94 (0.80–1.10)	0.458
Family income per month,¥			
<2000	as ref		
2000–4,000	**−0.24 (0.10)**	**0.78 (0.64–0.96)**	0.016*
4,000–6,000	**−0.65 (0.12)**	**0.52 (0.41–0.67)**	<0.001**
>6,000	**−0.73 (0.12)**	**0.48 (0.38–0.61)**	<0.001**
Source of income			
Acquiring from children	as ref		
Retirement income	−0.08 (0.09)	0.92 (0.76–1.11)	0.381
Labor income	−0.01 (0.14)	1.00 (0.76–1.30)	0.975
Education level			
Illiteracy	as ref		
Primary school and below	**0.56 (0.12)**	**1.75 (1.39–2.20)**	<0.001**
Junior high school	**2.45 (0.13)**	**11.61 (8.97–15.03)**	<0.001**
Senior high school	**2.17 (0.14)**	**8.72 (6.57–11.57)**	<0.001**
College and above	**1.99 (0.21)**	**7.33 (4.82–11.14)**	<0.001**
Type of insurance			
Rural medical service	as ref		
Urban medical service	**−0.21 (0.09)**	**0.81 (0.68–0.97)**	0.023*
Other medical service	−0.14 (0.15)	0.87 (0.65–1.17)	0.358
Place of residence			
Villages	as ref		
Counties or towns	0.09 (0.11)	1.10 (0.88–1.37)	0.414
Small or middle-sized cities	**0.41 (0.12)**	**1.50 (1.20–1.89)**	<0.001**
Large-sized cities	**0.53 (0.10)**	**1.71 (1.40–2.08)**	<0.001**
Cardiovascular or cerebrovascular diseases	0.19 (0.13)	1.21 (0.93–1.57)	0.156
Hypertension	0.06 (0.10)	1.07 (0.87–1.30)	0.538
Coronary heart disease	**0.23 (0.11)**	**1.26 (1.02–1.55)**	0.029*
Diabetes	0.13 (0.10)	1.14 (0.93–1.39)	0.200
Type of medication			
None	as ref		
1	0.01 (0.12)	1.01 (0.80–1.27)	0.967
2	**−0.45 (0.13)**	**0.64 (0.49–0.82)**	0.001**
3	**−0.48 (0.15)**	**0.62 (0.46–0.83)**	0.002**
>4	**−0.58 (0.15)**	**0.56 (0.42–0.76)**	<0.001**
Annual physical examination for the last 10 years	**0.21 (0.08)**	**1.24 (1.06–1.45)**	0.007**
Weekly social activities, days			
Never	as ref		
1–3	**−0.28 (0.09)**	**0.76 (0.63–0.91)**	0.003**
4–6	−0.27 (0.19)	0.77 (0.53–1.10)	0.152
Every day	**−0.32 (0.10)**	**0.73 (0.60–0.89)**	0.002**
Weekly exercise, days			
Never	as ref		
1–3	0.12 (0.10)	1.13 (0.93–1.38)	0.228
4–6	0.32 (0.18)	1.38 (0.96–1.98)	0.078
Every day	0.14 (0.10)	1.15 (0.95–1.41)	0.157
Support from family	−0.11 (0.08)	0.90 (0.77–1.05)	0.166
Care from family or friends	**−0.36 (0.10)**	**0.70 (0.57–0.85)**	<0.001**
Hearing	**0.21 (0.08)**	**1.24 (1.05–1.46)**	0.011*
Vision	0.16 (0.08)	1.17 (0.99–1.36)	0.052
Walking	**0.51 (0.09)**	**1.67 (1.38–2.02)**	<0.001**
Anxiety			
None	as ref		
Mild	−0.06 (0.11)	0.94 (0.76–1.17)	0.573
Moderate	−0.24 (0.23)	0.79 (0.51–1.23)	0.299
Moderate–severe	0.57 (0.30)	1.76 (0.98–3.17)	0.058
Severe	**1.16 (0.58)**	**3.17 (1.02–9.83)**	0.045*
Depression			
None	as ref		
Mild	**0.56 (0.12)**	**1.75 (1.39–2.19)**	<0.001**
Moderate	**0.93 (0.19)**	**2.54 (1.75–3.68)**	<0.001**
Moderate–severe	**1.34 (0.28)**	**3.80 (2.18–6.64)**	<0.001**
Severe	**1.81 (0.48)**	**6.08 (2.38–15.57)**	<0.001**
Physical function impairment	**0.58 (0.09)**	**1.79 (1.49–2.16)**	<0.001**

OR = odds ratio, 95% CI = 95% confidence interval.

*p < 0.05, **p < 0.01.

The statistically significant variables are highlighted as bold values.

Regarding behavior habits, older adults who had undergone annual physical examinations over the last decade (*OR*: 1.23, 95%*CI*: 1.05–1.44) and those who did not participate in weekly social activities were more likely to develop cognitive impairment. In terms of family or social support, older adults who were not cared for by family or friends were at a higher risk of cognitive impairment. Several health conditions were also found to be significantly associated with cognitive impairment, such as coronary heart disease (*OR*: 1.26, 95% *CI*: 1.03–1.55), hearing disorders (*OR*: 1.24 95% *CI*: 1.05–1.47), and impaired walking ability (*OR*: 1.67, 95% *CI*: 1.30–2.02). Furthermore, older adults who did not take any medication were more likely to experience cognitive impairment than those taking two or more types of medication. Regarding psychological factors, the results revealed that risk factors significantly associated with cognitive impairment were severe anxiety (*OR*: 3.16, 95% *CI*: 1.02–9.81) and depression. Moreover, as shown in Tables 3, 4, we found that physical function impairment (*OR*: 1.79, 95%*CI*: 1.49–2.16) and cognitive impairment (*OR*: 1.83, 95% *CI*: 1.51–2.21) were independent risk factors for each other among older adults.

## 4 Discussion

We found that physical function impairment existed in nearly one-third of subjects in the total population, with a prevalence of 37.3%. This result was similar to the data from Vásquez et al. (2022), who studied 3,050 older adults in the Hispanic Established Populations for Epidemiologic Study, and Farías-Antúnez et al. (2018), who investigated 1,451 older adults in Brazil. However, this rate of disability was significantly lower than that reported in a nationwide population-based longitudinal survey of healthy aging, which randomly selected participants from 22 provinces in China (Hou et al., 2019).

The prevalence of disability in the above study was more than half of the total number of individuals in both urban and rural areas. This is likely because the average age of the older adults included in our survey was generally younger. Moreover, several native regional studies close to Guangdong Province have reached similar results. For example, one population-based study (Chen et al., 2018) conducted in Guangxi Province involving 2,300 adults aged 60 years or older indicated that the disability rates, measured with ADL and IADL, were 43.4 and 42.4%, respectively.

A similar conclusion was reached in another study conducted in the northeastern rural areas of India that involved a community-based population (Medhi et al., 2021). The likely reason is that the urban and rural distribution in our study was almost balanced, whereas a higher proportion of participants in the aforementioned studies resided in rural areas.

The discrepancy in the evaluation criteria for BADL or IADL in the two studies may also lead to inconsistent results. To make the results more accurate and representative, future multicenter, large-scale epidemiological investigations should focus on uniform distributions of age and region during sampling and consistency of measurement methods.

The overall cognitive impairment rate in our study was 31%. This result supports the proportion of surveys conducted for representative older adults from Brazil (34.0%) (Brigola et al., 2020). However, many population-based cross-sectional studies carried out in different countries, such as Spain (Lara et al., 2016), Italy (Caffò et al., 2022), Mexico (Givan et al., 2022), Japan (Saw et al., 2020), and Korea (Lee et al., 2022), all showed a lower cognitive impairment rate. Nationwide data from China, based on samples of 46,011 (Jia et al., 2020), 21,732 (Qi et al., 2021), and 3,768 (Qin et al., 2022) in 2018, also showed prevalence rates of 15.0, 17.8, and 22.4%, respectively, which were significantly lower than those of our study. A similar result was observed in other epidemiological studies in eastern (Ding et al., 2015) and northern China (Jiang et al., 2021; Zhang et al., 2019).

Qin et al. (2022) also analyzed regional prevalence and found that the rate of cognitive impairment among older adults in the southwest region was 29.9%, the highest among all regions. These data are closely aligned with our results. The differences by country or region may be related to complex factors such as race, lifestyle, economic level, and medical practices. All findings suggest that southern China should be a major focus for preventing and controlling cognitive impairment or dementia. Further studies should emphasize developing interventions and management systems that consider regional characteristics.

The results of our study’s multivariate logistic regression analysis indicated that age, family income, education level, type of medication, physical examination, social activities, support from family or friends, hearing ability, walking, and depression were all associated with both physical function and cognitive impairment. There was broad consensus linking older age to disability, whether in terms of body or cognition (Chen et al., 2018; Goswami et al., 2019; Ramadass et al., 2018).

We also confirmed this view in previous studies. The possible reason may be that older adults often experience several irreversible declines in organic functions and degenerative changes, such as Alzheimer’s disease, as they age, which can directly impair self-care and daily living abilities. High family income was recognized as a significant protective factor against cognitive impairment in our study, aligning with most published research on (Danielewicz et al., 2019; Philibert et al., 2013) the relationship between socioeconomic status or income and the incidence of disability. This phenomenon may be explained by the fact that older adults with low family incomes often lack sufficient financial resources and social support to cope with increased healthcare burdens, have less access to medical services for chronic disease management, and experience restricted interaction with their social networks, which contributes to the development of functional disorders (Bowling and Stafford, 2007; Zeng et al., 2010; Vaughan et al., 2016). There was a significant difference in the prevalence of physical and cognitive impairments among older adults with varying education levels. A higher level of education was linked to a reduced risk of physical function impairment, while the opposite was observed for cognitive impairment.

Previous studies (Goswami et al., 2019; Ravi et al., 2022) tended to support the viewpoint that education level is a protective factor, as individuals with more education have greater access to resources and knowledge for health-related services, enhancing their ability to manage diseases. However, in our study, more educated older adults exhibited a higher risk of cognitive impairment, which is inconsistent with the majority of studies (Xue et al., 2022; Liu et al., 2021; Wang et al., 2020) investigating the relationship between cultural features and disability.

However, the findings of Godinho et al. (2022) are consistent with our findings, which may be explained by complex neuropathologic theories and structural and functional changes in brain features due to aging. Further research into mechanistic exploration is needed to better interpret this relationship.

Our study found a positive association between medication use and physical function impairment, which aligns with previous findings (Chen et al., 2018; Medhi et al., 2021). Unfortunately, the opposite result was observed regarding cognitive aspects. One possible reason could be that older adults taking multiple types of medication may have various chronic diseases, leading them to seek medical assistance more frequently; thus, they might detect and manage the risk and early stages of cognition-related issues sooner.

Although the habit of undergoing regular physical examinations is beneficial for health promotion, the results of our study indicated that having an annual physical examination for the past 10 years was associated with a higher risk of cognitive impairment. We clarified that this phenomenon differed from conventional findings, as older adults who usually participate in physical examinations may have more health issues, which can lead to an increased risk of negative emotions or mental health problems, thereby accelerating cognitive decline (Freire et al., 2017; Desai et al., 2021).

The findings in our study also indicated that the effect of spiritual level support on disability, whether physical or cognitive, was more significant than that of material support, which highlighted the potential value of disability interventions based on psychological theories.

Over the last few years, some researchers have conducted large-scale population-based observational studies (Fuller-Thomson et al., 2022; Borges et al., 2021)and a longitudinal study (He et al., 2023) that explored the relationship between sensory disorders and cognitive impairment, which indicated that hearing disorders are an independent influencing factor on cognitive impairment. Our findings also align with this result. The reason for the observed correlation between sensory function and cognitive impairment may relate to potential mechanisms based on several hypotheses regarding internal effects, such as sensory deprivation (Lin et al., 2014; Nixon et al., 2019)and resource allocation (Nixon et al., 2019), or external effects, such as social disengagement (Fuller-Thomson et al., 2022). Unfortunately, there is no evidence to determine whether the relationship between sensory disorders and cognitive impairment is causal, nor to clarify the exact mechanisms, which require further longitudinal studies with large sample sizes or basic research for confirmation.

However, it may be necessary to pay particular attention to older adults with age-related sensory disorders, identify them, and provide early interventions to mitigate cognitive decline. Our study also suggested that elderly adults with walking disorders had a higher risk of impairments in physical and cognitive function. One systematic review (Binotto et al., 2018) included 49 studies that considered gait speed as a predictor of physical frailty and health indicators, showing a potential correlation between walking problems and disability that is consistent with our study.

The results of our study showed a significant association between psychological factors, such as anxiety and depression, and cognitive impairment. There is a broad consensus that psychological distress is an independent predictor of cognitive health (Freire et al., 2017; Desai et al., 2021). Therefore, investigating coping strategies for stress and developing intervention networks based on the social-psychological aspects of cognitive health among older adults may gradually become a key direction for future studies. Interestingly, we also found that depression was a contributing factor to physical function impairment. While this finding aligns with numerous previous studies (Kang et al., 2017; Taş et al., 2007), the connection between these two variables remains debatable (Duba et al., 2012) and merits further discussion.

In addition to the factors mentioned above, the opposite result obtained in our study, that smoking was a protective factor against disability, was unusual compared to most studies addressing the negative effects of unhealthy lifestyles on diseases (Ravi et al., 2022; Ramadass et al., 2018; Medhi et al., 2021). This may be explained by the complex relationship between praxeology or psychology and diseases, but further studies are needed to investigate the validity of this result.

Interestingly, our study’s additional findings demonstrated that older adults with physical function impairment faced an increased risk of cognitive impairment; conversely, cognitive impairment also accelerated the progression of physical disability. Most published studies (Kiiti et al., 2019; Chong et al., 2015; Boyle et al., 2010; Shimada et al., 2013) suggest a strong link between physical and cognitive impairment, indicating that the association is bidirectional. Longitudinal studies (Chong et al., 2015; Boyle et al., 2010) have confirmed that physical function impairment or frailty is a predictor of cognitive decline among people with mild cognitive impairment and is associated with a higher risk of dementia.

Cognitive impairment is a potentially modifiable risk factor for physical disability in aging, impacting self-care and mobility. Therefore, interventions designed to slow or manage the progression of physical function impairment may be crucial in preventing cognitive impairment or dementia; conversely, the reverse strategy is also possible.

Our findings suggest that comprehensive interventions be implemented for screening and preventing disability to support healthy aging in China. The implications for public health policy and the medical system include the following: First, governments and healthcare administrations should pay more attention to regional discrepancies in disability, improve screening measures for older adults, and provide social security and healthcare services that meet health requirements. Second, healthcare managers must conduct early assessments and monitor functional status, developing intervention strategies for various functional impairments based on the characteristics of older adults, with the aim of delaying the disabling process. Finally, interventions related to physical and cognitive training for older adults should encompass the entire continuum of disease management and offer more accessible platforms and opportunities for family involvement, as this may effectively promote the reasonable allocation of medical resources.

To the best of our knowledge, this was the first large-scale population-based cross-sectional survey on the incidence of physical and cognitive impairment among older adults in the southern region of China. We collected comprehensive information on older adults via a digital platform, analyzed the prevalence of physical and cognitive impairment, and investigated the factors influencing disability. However, this study has certain limitations. First, as a cross-sectional study, it cannot verify the causal association between the included factors and physical function or cognitive impairment. Second, physical function can be classified in ways other than by BADL or IADL, such as muscle strength, balance, and gait speed, but we did not perform other measurements beyond the method of questionnaires due to restrictions on instruments and the population base. Third, the MMSE is merely a cognitive screening tool and cannot clarify the diagnosis of cognitive impairment and its subtypes. Further studies need to use more nuanced measures to assess cognitive function across various domains, thereby verifying the conclusions of the present study. Finally, some results of this study might be biased because older adults with hearing or vision loss cannot complete the survey themselves and require assistance from caregivers or family members, which may introduce subjective opinions from proxy respondents affecting the data. Participants’ ability to recall past information may also lead to inaccurate results.

## 5 Conclusion

The findings from this population-based cross-sectional study in Guangdong Province demonstrated a high incidence rate of disability in terms of physical dysfunction and cognitive impairment. Numerous influencing factors related to age, family income, education level, type of medication, physical examination, social activities, support from family or friends, hearing or walking disorders, and depression were linked to declines in physical or cognitive function. Physical dysfunction was significantly correlated with cognitive impairment among older adults.

## Data Availability

The raw data supporting the conclusions of this article will be made available by the authors, without undue reservation.
